# Supplementary material for: Mouse MRE11-RAD50-NBS1 is needed to start and extend meiotic DNA end resection
Source: Nat Commun. 2025 Apr 16;16:3613. doi: 10.1038/s41467-025-57928-x (PMC12003770; doi:10.1038/s41467-025-57928-x)
Supplement: Supplementary file 2 — Description of Additional Supplementary Files [file 41467_2025_57928_MOESM2_ESM.pdf]

File name: Supplementary Data 1
Description: Excel file containing S1-seq, Exo7/T-seq and MRE11-ChIP mapping statistics.

File name: Supplementary Data 2.
Description: Excel file listing primary and secondary antibodies used in this study.
