# Supplementary material for: Mouse MRE11-RAD50-NBS1 is needed to start and extend meiotic DNA end resection
Source: Nat Commun. 2025 Apr 16;16:3613. doi: 10.1038/s41467-025-57928-x (PMC12003770; doi:10.1038/s41467-025-57928-x)
Supplement: Supplementary file 3 — Reporting Summary [file 41467_2025_57928_MOESM3_ESM.pdf]

nature portfolio

Corresponding author(s): Scott Keeney

Last updated by author(s): Feb 24, 2025

Reporting Summary

Nature Portfolio wishes to improve the reproducibility of the work that we publish. This form provides structure for consistency and transparency in reporting. For further information on Nature Portfolio policies, see our Editorial Policies and the Editorial Policy Checklist.

Statistics

For all statistical analyses, confirm that the following items are present in the figure legend, table legend, main text, or Methods section.

n/a	Confirmed	
☐	☒	The exact sample size (*n*) for each experimental group/condition, given as a discrete number and unit of measurement
☐	☒	A statement on whether measurements were taken from distinct samples or whether the same sample was measured repeatedly
☐	☒	The statistical test(s) used AND whether they are one- or two-sided<br>*Only common tests should be described solely by name; describe more complex techniques in the Methods section.*
☒	☐	A description of all covariates tested
☒	☐	A description of any assumptions or corrections, such as tests of normality and adjustment for multiple comparisons
☐	☒	A full description of the statistical parameters including central tendency (e.g. means) or other basic estimates (e.g. regression coefficient) AND variation (e.g. standard deviation) or associated estimates of uncertainty (e.g. confidence intervals)
☐	☒	For null hypothesis testing, the test statistic (e.g. *F*, *t*, *r*) with confidence intervals, effect sizes, degrees of freedom and *P* value noted<br>*Give P values as exact values whenever suitable.*
☒	☐	For Bayesian analysis, information on the choice of priors and Markov chain Monte Carlo settings
☒	☐	For hierarchical and complex designs, identification of the appropriate level for tests and full reporting of outcomes
☒	☐	Estimates of effect sizes (e.g. Cohen's *d*, Pearson's *r*), indicating how they were calculated

Our web collection on statistics for biologists contains articles on many of the points above.

Software and code

Policy information about availability of computer code

Data collection	Conventional microscopy image acquisition: Marianas Slidebook (version 5.0)<br>Structured illumination microscopy image acquisition: Deltavision softWoRx (version 7.0.0)<br>Whole histology slide scan: Panoramic Flash Slide Scanner (version 3.2.0) and Zeiss Axio Imager (version 3.2).<br>Chemiluminescence western blotting scan: Image Lab (version 6.1.0 build 7)<br>Phosphor Imaging scan: Amersham typhoon control software (version 4.0.0.4)<br>Illumina sequencing: Real Time Analysis (version 3.1 or version 4.1) or HiSeq Control Software (version 2.2.68)
Data analysis	ImageJ Fiji (version 1.54g) or CaseViewer (version 2.4) was used for image processing.<br>Sequencing Base calls: bcl-convert software (version 3.9.3 to 3.10.5) or bcl2fastq software (version 2.20)<br>Trim Galore (version 0.6.6)<br>bowtie2 (version 2.3.5.1)<br>R (versions 4.0.3 to 4.3.2) and R studio (version2023.06.1+524)<br>GraphPad Prism 9 or 10.<br>Final figure assembly using Adobe illustrator 2023 or 2024.

For manuscripts utilizing custom algorithms or software that are central to the research but not yet described in published literature, software must be made available to editors and reviewers. We strongly encourage code deposition in a community repository (e.g. GitHub). See the Nature Portfolio guidelines for submitting code & software for further information.

Data

Policy information about availability of data

All manuscripts must include a data availability statement. This statement should provide the following information, where applicable:

- Accession codes, unique identifiers, or web links for publicly available datasets
- A description of any restrictions on data availability
- For clinical datasets or third party data, please ensure that the statement adheres to our policy

Raw and processed sequencing S1-seq, Exo7/T-seq, MRE11-ChIP, and SSDS data have been deposited in the Gene Expression Omnibus (GEO) repository under accession numbers GSE266258 [https://www.ncbi.nlm.nih.gov/geo/query/acc.cgi?acc=GSE266258], GSE266151 [https://www.ncbi.nlm.nih.gov/geo/query/acc.cgi?acc=GSE266151], and GSE266271 [https://www.ncbi.nlm.nih.gov/geo/query/acc.cgi?acc=GSE266271]. We used additional S1-seq and Exo7/T-seq data from GEO accession numbers GSE265863 [https://www.ncbi.nlm.nih.gov/geo/query/acc.cgi?acc=GSE265683] and GSE229450 [https://www.ncbi.nlm.nih.gov/geo/query/acc.cgi?acc=GSE229450] 34,117. We used SPO11-oligo data from GEO accession numbers GSE84689 [https://www.ncbi.nlm.nih.gov/geo/query/acc.cgi?acc=GSE84689] and Atm–/– MRE11 ChIP-seq data from GEO accession GSE138915 [https://www.ncbi.nlm.nih.gov/geo/query/acc.cgi?acc=GSE138915] 14,37. Code used for read processing and mapping is available online at https://github.com/yamadas2/mouse-S1seq, which also includes scripts for generating mean profiles and heatmaps around SPO11-oligo hotspot centers, and scatter plots to check correlation with SPO11-oligo maps and reproducibility between maps as previously described34. The mouse genome assembly mm10 (also known as GRCm38) is available at https://www.ncbi.nlm.nih.gov/datasets/genome/GCF_000001635.20/. Source data are provided with this paper.

Research involving human participants, their data, or biological material

Policy information about studies with human participants or human data. See also policy information about sex, gender (identity/presentation), and sexual orientation and race, ethnicity and racism.

Reporting on sex and gender	n/a, no human subjects data
Reporting on race, ethnicity, or other socially relevant groupings	n/a, no human subjects data
Population characteristics	n/a, no human subjects data
Recruitment	n/a, no human subjects data
Ethics oversight	n/a, no human subjects data

Note that full information on the approval of the study protocol must also be provided in the manuscript.

Field-specific reporting

Please select the one below that is the best fit for your research. If you are not sure, read the appropriate sections before making your selection.

[x] Life sciences [ ] Behavioural & social sciences [ ] Ecological, evolutionary & environmental sciences

For a reference copy of the document with all sections, see nature.com/documents/nr-reporting-summary-flat.pdf

Life sciences study design

All studies must disclose on these points even when the disclosure is negative.

Sample size	No sample size calculations were performed. All sample sizes were chosen based on established best practices in the field for the experimental methods used. All experiments were performed at least twice unless stated otherwise. Sequencing experiments were conducted in two biological replicates unless stated otherwise, because two replicates are sufficient to determine reproducibility because of the high correlation between replicates for these methods (references: this study and PMID 39149289 and PMID 32354835). For S1-seq of Rad50S (Extended data Fig. 4o), technical replicates were done to reduce animal breeding for mutant mice When reproducing already published sequencing results, a single experiment was conducted and reproducibility was confirmed by comparison with published data.
Data exclusions	For sequencing data: sequence reads mapping to repetitive DNA were excluded because they cannot be assigned with confidence to specific locations, and our previous published work has established that excluding them does not affect the conclusions drawn (PMID 32354835). No other data were excluded.
Replication	All attempts at replication were successful. All conclusions described in the paper were based on findings reproduced in biological replicate experiments except for Fig 2f, which was performed once.
Randomization	Not relevant: All experiments involved comparison of control (wild type or other appropriate controls) and mutant mouse strains, so randomization is neither necessary nor appropriate.
Blinding	No blinding was used. All results presented in the paper involved side-by-side comparison of mutants with appropriate controls. For molecular cytology experiments, blinding was not possible as mutant mouse strains used in this study display distinct and specific characteristics such as abnormal axis structure. Mutational effects in sequencing experiments had large effect size relative to the precision of the methods and

relative to variation that could be ascribed to operator bias, so operator bias is not a relevant consideration for this experimental design.

Reporting for specific materials, systems and methods

We require information from authors about some types of materials, experimental systems and methods used in many studies. Here, indicate whether each material, system or method listed is relevant to your study. If you are not sure if a list item applies to your research, read the appropriate section before selecting a response.

Materials & experimental systems

n/a	Involved in the study
☐	☒ Antibodies
☐	☒ Eukaryotic cell lines
☒	☐ Palaeontology and archaeology
☐	☒ Animals and other organisms
☒	☐ Clinical data
☒	☐ Dual use research of concern
☒	☐ Plants

Methods

n/a	Involved in the study
☐	☒ ChIP-seq
☒	☐ Flow cytometry
☒	☐ MRI-based neuroimaging

Antibodies

Antibodies used

Primary antibodies: mouse anti-SYCP3 (Santa Cruz, sc-74569), rabbit anti-SYCP1 (Abcam, ab15090), rabbit anti-RPA2 (Abcam, ab76420), rabbit anti-DMC1 (Santa Cruz, sc-22768), mouse anti-DMC1 (Abcam, ab11054), guinea pig anti-DMC1(Hinch et al., 2020, home-made T113-1, ABclonal Biotechnology), rabbit anti-RAD51 (Santa Cruz, sc-8349), mouse anti-RAD51 (Novus Biologicals, NB100-148), rabbit anti-γH2AX (Abcam, ab2893), rat anti-tubulin alpha (Bio-Rad, MCA78G), rabbit anti-MRE11 (Novus Biologicals, NB100-142 and Yu et al., 2012, custom made), rabbit anti-RAD50 (Novus Biologicals, NB100-154 and Yu et al., 2012, custom made), rabbit anti-NBS1 (Novus Biologicals, NB100-154 and Yu et al., 2012, custom made), mouse anti-KAP1 (Santa Cruz, sc-136102), rabbit anti-pKAP1 Ser824 (Abcam, ab70369), mouse anti-beta actin (Abcam, ab49900), rabbit anti-pHistone H3 Ser10 (Millipore, 06-570), mouse anti-SPO11 (Lange et al., 2011, home-made, clone 180, MSKCC Antibody and Bioresource Core Facility), rabbit anti-DDX4 (Abcam, ab13840). Secondary antibodies: Alexa-488 goat anti-guinea pig (Invitrogen, A-11073), CF405S goat anti-mouse (Biotium, BT20080), Alexa-594 goat anti-mouse (Invitrogen, A-11005), Alexa-555 donkey anti-rabbit (Invitrogen, A-31572).

Validation

Antibodies from Santa Cruz and Abcam were all previously used for immunofluorescence in various publications. Antibodies from Novus Biologicals were all previously used for western blotting in various publications. All home-made antibodies were characterized in previous publications as cited above. Antibodies against MRE11 (Novus Biologicals) were recommended for ChIP-seq in mammalian cells (Wienert et al., 2020) but haven't been tested for mouse testis tissue. In this study, we validated this MRE11 antibody for ChIP-seq from Atm-/- mouse testis and reproduced previously published data (Paiano et al., 2020). This antibody was further tested for immunohistochemstry on mouse testis sections and specificity was confirmed by loss or alteration of signal in Mre11 mutants. Antibodies against DMC1 (Abcam) were tested ChIP followed by single-stranded DNA sequencing and produced consistent results as previous publications (Khil et al., 2012 and Hinch et al., 2020).

Eukaryotic cell lines

Policy information about cell lines and Sex and Gender in Research

Cell line source(s)	Mouse embryonic fibroblast (MEF) cell lines were derived from E13.5 un-sexed mouse embryos from the Petrini lab colony.
Authentication	The cell lines were not authenticated.
Mycoplasma contamination	Cell lines were not tested for mycoplasma.
Commonly misidentified lines (See ICLAC register)	None.

Animals and other research organisms

Policy information about studies involving animals; ARRIVE guidelines recommended for reporting animal research, and Sex and Gender in Research

Laboratory animals

Only male mice were used because this study focused on spermatogenesis. Mice were housed in solid-bottom, polysulfone, individually ventilated cages (IVCs) (Thoren Caging Systems, Hazelton, PA) on autoclaved aspen-chip bedding (PWI Industries Canada, Quebec, Canada); γ-irradiated feed (LabDiet 50531, PMI, St Louis, MO) and acidified reverse osmosis water (pH 2.5 to 2.8) provided ad libitum. The cages also contained Nestlets®, EnviroDri®, and/or EnviroPaks® as environmental enrichment. The IVC system was ventilated at approximately 30 air changes hourly. HEPA-filtered room air was supplied to each cage and the rack effluent was exhausted directly into the building's exhaust system. Cages were changed weekly in either a HEPA-filtered vertical flow change station or a Class 2 Type A biological safety cabinet. The animal holding room was maintained at 21.5 ± 1 °C, relative humidity between 30% and 70%, and a 12:12 hour light:dark photoperiod. Mice were euthanized by CO_2 asphyxiation prior to tissue harvest.

Wild animals	The study did not involve wild animals
Reporting on sex	Experiments analyzed spermatogenesis, so only male mice were used.
Field-collected samples	The study did not involve samples collected from the field.
Ethics oversight	Experiments conformed to the US Office of Laboratory Animal Welfare regulatory standards and were approved by the Memorial Sloan Kettering Cancer Center Institutional Animal Care and Use Committee (protocol numbers 01-03-007 and 01-12-034).

Note that full information on the approval of the study protocol must also be provided in the manuscript.

Plants

Seed stocks	*Report on the source of all seed stocks or other plant material used. If applicable, state the seed stock centre and catalogue number. If plant specimens were collected from the field, describe the collection location, date and sampling procedures.*
Novel plant genotypes	*Describe the methods by which all novel plant genotypes were produced. This includes those generated by transgenic approaches, gene editing, chemical/radiation-based mutagenesis and hybridization. For transgenic lines, describe the transformation method, the number of independent lines analyzed and the generation upon which experiments were performed. For gene-edited lines, describe the editor used, the endogenous sequence targeted for editing, the targeting guide RNA sequence (if applicable) and how the editor was applied.*
Authentication	*Describe any authentication procedures for each seed stock used or novel genotype generated. Describe any experiments used to assess the effect of a mutation and, where applicable, how potential secondary effects (e.g. second site T-DNA insertions, mosiacism, off-target gene editing) were examined.*

ChIP-seq

Data deposition

☒ Confirm that both raw and final processed data have been deposited in a public database such as GEO.

☐ Confirm that you have deposited or provided access to graph files (e.g. BED files) for the called peaks.

Data access links<br>*May remain private before publication.*	All sequencing data are publicly available (see Data section above for links). No peak calling was performed for the ChIP-seq experiments.
Files in database submission	All raw and processed files were submitted to GEO.
Genome browser session<br>(e.g. UCSC)	*Provide a link to an anonymized genome browser session for "Initial submission" and "Revised version" documents only, to enable peer review. Write "no longer applicable" for "Final submission" documents.*

Methodology

Replicates	MRE11 ChIP-seq for wild type was conducted in two biological replicates and for Mre11 nuclease dead was conducted in three biological replicates. MRE11 ChIP-seq for Atm-/- was done once and compared with published results.<br>DMC1 ChIP-SSDS for wild type was done once and compared with published results. All other SSDS experiments were conducted in two biological replicates.
Sequencing depth	Sequencing and mapping statistics are provided in Supplementary Table S2
Antibodies	Anti-MRE11 (Novus Biologicals, NB100-142), anti-DMC1 (Abcam, ab11054), anti-RAD51 (Novus Biologicals, NB100-148), anti-RPA2 (Abcam, ab76420).
Peak calling parameters	Peak calling was not performed
Data quality	Data quality was assessed by analyzing reproducibility of replicates. No peak calling was performed.
Software	Base calls were perfomed using bcl2fastq software v.2.20 or bcl-convert software v.3.9.3 to 3.10.5 for samples sequenced on Illumina NovaSeq 6000. Reads were trimmed and filtered by Trim Galore version 0.6.6 with the arguments --paired --length 15. Sequence reads were mapped onto the mouse reference genome (mm10) by bowtie2 version 2.3.5.1 with the arguments -N 1 -X 1000. Duplicated reads were removed by Picard. Uniquely and properly mapped reads (MAPQ ≥ 20) were extracted by samtools with the arguments -q 20.
